# Biochar and *Bacillus subtilis* co-drive dryland soil microbial community and enzyme responses

**DOI:** 10.3389/fmicb.2025.1603488

**Published:** 2025-05-27

**Authors:** Tao Zheng, Xianhuai Huang, Xiaoyu Zhou, Jizi Wu, Muhammad Aqeel Kamran, Xiongsheng Yu, Jing Qian

**Affiliations:** ^1^School of Environment and Energy Engineering, Anhui JianZhu University, Hefei, China; ^2^Anhui Provincial Key Laboratory of Environmental Pollution Control and Resource Reuse, Hefei, China; ^3^Hu Zhou Shi Zhi Bao Jian Yi Yu Geng Fei Guan Li Zhan, Huzhou, China; ^4^College of Environmental and Resource Sciences, Zhejiang A&F University, Lin'an, China; ^5^Xishuangbanna Tropical Botanical Garden Yunnan Province, Mengla, Yunnan, China; ^6^Department of Chemistry, Xinzhou Normal University, Xinzhou, Shanxi, China

**Keywords:** biochar, *Bacillus subtilis*, dryland soil, bacterial community, fungal communities

## Abstract

**Introduction:**

To investigate the impact of soil amendments on the structure of the soil microbial community.

**Methods:**

This study focuses on dryland soil and employs indoor static cultivation as the experimental approach. It analyzes the impact and mechanism of adding rice straw biochar (S), rapeseed straw biochar (Y), and *Bacillus subtilis* agent (J) separately and in combination on the soil microbial community structure.

**Results:**

The experimental results indicated that, compared to the blank control (CK), the Y treatment increased the relative abundance of *Proteobacteria* by approximately 3.03% and significantly reduced the abundance of *Acidobacteria* (from 70.56% to 82.81%). The application of biochar and microbial inoculants significantly increased the relative abundance of *Ascomycota* (2.85% to 33.53%) and *Rozellomycota* (0.58% to 27.73%). Furthermore, the addition of soil amendments enhanced the richness (3.02% to 7.07%) and diversity (3.22% to 3.77%) of soil bacteria, as well as the microbial nitrogen content (3.7 to 9.3 times). Meanwhile, except for the YJ treatment, the richness of the fungal community decreased, while the diversity index increased. The experimental results showed that the application of rapeseed straw biochar or the compound microbial inoculant alone significantly increased soil urease activity, reaching 40.34 µg of NH^+^_4_-N g^−1^ of soil h^−1^ and 40.29 µg of NH^+^_4_-N g^−1^ of soil h^−1^ at the end of the incubation period, respectively.

**Discussion:**

In conclusion, rapeseed straw biochar not only enhances the soil microbial community but also significantly influences soil enzyme activity. This study offers a scientific foundation for utilizing biochar and *Bacillus subtilis* to improve dryland soil, providing valuable insights for sustainable soil management.

## 1 Introduction

Drylands, constituting a critical agricultural land use category in China, encompassed over 6.4 × 10^5^ km^2^ by 2020, accounting for more than half of the total arable land area in the country (Zhao H. F. et al., 2024). Recent decades have witnessed progressive soil quality deterioration in these ecosystems, driven by sustained high-intensity cultivation practices and excessive agrochemical inputs. This degradation manifests as diminished soil fertility (Li et al., 2023) accelerated nutrient leaching, and exacerbated eutrophication of adjacent aquatic systems. These cumulative effects pose dual threats to environmental integrity and agricultural productivity, ultimately constraining sustainable socioeconomic development in rural regions (Liu and Pu, 2019). Consequently, implementing targeted interventions to restore pedological functions, rehabilitate nutrient cycling capacity, and boost agricultural productivity has become imperative for addressing these interconnected agro-environmental challenges. Notably, cost-effective and environmentally friendly soil amendments have proven effective in improving soil properties through a dual mechanism of fertility enhancement and structural optimization. This combined functionality has accelerated their adoption as sustainable solutions in modern agricultural systems.

Biochar, a byproduct of the pyrolysis of waste biomass resources under high temperatures and limited oxygen conditions, is an environmentally friendly material commonly used for soil improvement and optimization of soil structure. Previous studies have shown that the addition of biochar can effectively enhance soil fertility and increase crop yields. For example, Fan et al. (2024) used Rosa Roxburghe pomace biochar to amend sandy clay loam soil, which increased soil pH, organic matter, and available nutrient content by 41.06, 134.84, and 341.75 to 627.13%, respectively. It also enhanced the activity of urease (by 51.43 to 362.86%) and catalase (by 21.40 to 85.12%). Biochar, with its rich porous structure and nutrient content, provides a favorable environment for microbial life. Additionally, the application of biochar to soil can effectively improve soil aggregate structure, alleviate soil compaction, and enhance soil water retention and aeration. These improvements, in turn, stimulate soil microbial activity and the secretion of extracellular enzymes (Huang et al., 2017). By altering relevant soil environmental factors, biochar can indirectly change the structure of microbial communities, such as inducing changes in bacterial communities like *Actinobacteria* and *Firmicutes* (Ren et al., 2020). In the study by Yu G. et al. (2024) the addition of biochar increased the relative abundance of *Actinobacteria* by 58.03%, optimized the structure of the soil bacterial community, and enhanced soil organic matter synthesis and carbon sequestration. Su et al. (2025) investigated the substitution of conventional fertilizers with microalgae and microbial inoculants, demonstrating that this amendment strategy significantly altered rhizobacterial community composition, stimulated carbon-cycling taxa, and improved soil stoichiometry through microbial community restructuring, ultimately enhancing *Polygala tenuifolia* biomass production. In the study by Zhang et al. (2017) the application of corn straw biochar to acidic soil increased soil pH and cation exchange capacity (CEC), enhanced the abundance of beneficial microorganisms such as *Bacillus* and *Bacteroidetes* and reduced the relative abundance of the pathogenic *bacterium Ralstonia solanacearum* by 94.51%. However, different types of biochar may have significantly different impacts on soil microbial community structure. Ji et al. (2022) pointed out that biochar with a high lignin content is conducive to the growth of *Gram-negative bacteria*, while manure biochar, which has a high ash content, is more favorable for the proliferation of nutrient-rich microorganisms (Wei et al., 2019).

Microbial inoculants are also a widely used soil amendment. Among them, *Bacillus subtilis*, a plant growth-promoting rhizobacterium (PGPR), has achieved good results in improving soil pests and diseases and promoting plant growth. Yu Y. et al. (2024) the application of *Bacillus subtilis* to the soil of mulberry trees has been found to significantly increase the alkaloid content in their tender leaves. Additionally, Sun et al. (2020) replaced 50% of the urea in sandy soil planted with wheat and corn with biofertilizer containing *Bacillus subtilis*, resulting in a 54% reduction in nitrogen loss and a 5% increase in crop yield. Wu et al. (2020) conducted a comparative field experiment evaluating lime versus biochar amendments on soil amelioration and *Citrus reticulata* yield. Their results revealed that biochar amendments exhibited superior capacity in enhancing soil enzymatic profiles, particularly urease and catalase activities, compared to lime treatments. This microbial-functional enhancement correlated with improved fruit yield metrics, suggesting biochar’s potential as a multifunctional soil conditioner. The application of *Bacillus subtilis* formulations can also impact the structure of soil microbial communities and improve the activity of soil nutrients. For instance, Zhao et al. (2021) found that both single and co-inoculation of *Rhodopseudomonas palustris* and *Bacillus subtilis* in paddy soil significantly increased rice yields (by 9.84 to 17.73%) and promoted the relative abundance of plant-growth-promoting microbial groups such as *Bacteroidetes* and *Proteobacteria*.

However, the effect of single amendment treatments is often unstable, while combined treatments can stabilize or synergistically enhance their amelioration effects. For instance, Yang et al. (2023) found in greenhouse tomato cultivation that the application of microbial inoculants alone did not significantly increase the content of vitamin C and soluble sugars in tomatoes. However, when combined with biochar, the promoting effect of microbial inoculants on tomato growth was enhanced. Rékási et al. (2019) applied biochar and microbial inoculants to calcareous acidic sandy soil and found that the amendments effectively increased the pH of the acidic soil and enhanced the availability of soil phosphorus and potassium by 53 and 80%, respectively. Additionally, the infection of *arbuscular mycorrhizal fungi* was reduced by 70%. Chen W. et al. (2023) used *Bacillus subtilis* as a bio-fertilizer combined with biochar in a pot experiment. As a result, the incidence of radish wilt decreased by about 60%, and the harmful effects of traditional agricultural fertilizers were avoided. Javeed et al. (2022) demonstrated that coupling rice husk and wood chip biochar with *Bacillus subtilis* enhanced the availability of nutrients in sandy soil. In the study by Qi et al. (2021), it was found that the co-inoculation of corn straw biochar and microbial inoculants increased the Chao 1 index and Shannon index of soil microorganisms by 16.05 and 28.83%, respectively. This indicates that the combined application of biochar and microbial inoculants has a significant impact on soil microbial communities, enhancing the richness and diversity of soil microorganisms and improving soil community structure. In addition to this, microorganisms are an important component of the soil ecosystem and are the main drivers of soil nutrient cycles. They directly or indirectly affect the transformation and cycling of soil nutrients through competition, symbiosis, and other means (Fierer, 2017). Soil bacteria and fungi are the two main groups of soil microorganisms. Their distribution, structure, and changes in abundance have a more profound impact on soil quality and nutrient cycling. Therefore, clarifying the effects of biochar and *Bacillus subtilis* on soil bacteria and fungi, as well as their interactions, is crucial for soil improvement.

In summary, this study investigates the effects of different types of biochar and the combined application of *Bacillus subtilis* on soil enzyme activity, microbial community structure, and their underlying mechanisms. Using dryland soil as the research subject, a 90-day indoor constant-temperature static incubation experiment was conducted. Soil pH, microbial biomass carbon (MBC), microbial biomass nitrogen (MBN), enzyme activity, and high-throughput sequencing were analyzed to: (1) evaluate the effects of biochar and *Bacillus subtilis*, both individually and in combination, on microbial carbon and nitrogen dynamics; (2) assess their influence on soil enzyme activity; and (3) examine shifts in the composition and diversity of the soil microbial community. This study aims to enhance soil quality and microbial community structure through biochar and microbial amendments, providing a scientific foundation for sustainable soil management and improvement strategies.

## 2 Materials and methods

### 2.1 Preparation of materials

The incubation experiment was conducted from September to December 2023. The soil was collected from the surface layer (0–20 cm) of newly cultivated peanut land in Tangjiagang, WuXing District (30°48′32″N, 120°11′07″E). The area has a subtropical monsoon climate, with an average annual temperature of 12.2–17.3°C, a frost-free period of 224–246 days, an average annual precipitation of about 761–1780 mm, and an average annual sunshine duration of 1,613–2,430 h. The surface soil (0–20 cm) was collected using the “S” shaped sampling method. The collected soil samples were thoroughly mixed, air-dried, and then sieved through a 2 mm mesh to remove stones and plant roots. The soil was then stored for later use. The physical and chemical properties of the test soil are detailed in Table 1.

**Table 1:** Physical and chemical properties of experimental soil.

Soil types	pH	Clay(%)	Silt(%)	Sand(%)	TN (g kg^−1^)	DOC (mg kg^−1^)	TC (g kg^−1^)	AK (mg kg^−1^)	AP (mg kg^−1^)
Infiltration of paddy soil(Dryland)	5.99 ± 0.15	15.15	33.14	51.71	1.02 ± 0.17	272.37 ± 16.4	11.35 ± 0.56	0.31 ± 0.03	52.03 ± 4.2

TN, total nitrogen; DOC, dissolved organic carbon; TC, total carbon; AK, available potassium; AP, available phosphorus.

The biochar used in this experiment included rapeseed straw biochar and rice straw biochar, both sourced from Nanjing QinFeng Straw Technology Co., Ltd., Jiangsu Province. The biochar preparation process involved crushing rapeseed and rice straw using a mechanical grinder, followed by air-drying and sieving through a 60-mesh screen. The preprocessed materials were then subjected to pyrolysis in a tube furnace at 600°C under a nitrogen (N₂) atmosphere for 2 h, with a controlled heating rate of 5°C/min. After cooling, the biochar was finely ground and passed through a 100-mesh sieve. To remove impurities, it was repeatedly washed with distilled water, dried at 105°C to a constant weight, and subsequently sealed for storage. For reference, rapeseed straw biochar was designated as Y, while rice straw biochar was labeled as S. The detailed physical and chemical properties of the biochar are presented in Table 2.

**Table 2:** Physical and chemical properties of experimental biochar.

Types of biochar	N (%)	C (%)	H (%)	S (%)	pH	Specific surface area (m^2^ g^−1^)	C/N
Rape biochar (Y)	0.875 ± 0.09	58.6 ± 0.11	2.088 ± 0.06	0.533 ± 0.06	9.52 ± 0.14	3.21 ± 0.33	66.97
Rice biochar (S)	2.29 ± 0.07	47.565 ± 0.09	1.7645 ± 0.16	0.235 ± 0.05	9.44 ± 0.11	1.54 ± 0.17	20.77

### 2.2 Experimental design

The experiment was conducted using a static indoor incubation method, Experimental treatments were designed based on established protocols from previous pedological investigations (Deng et al., 2025; Wang et al., 2025), and a total of six treatments were established: (1) CK (control), (2) Y, 4% rapeseed straw biochar (on a mass basis), (3) YJ, 4% rapeseed straw biochar + 5 mg kg^−1^ microbial agent, (4) S, 4% rice straw biochar, (5) SJ, 4% rice straw biochar + 5 mg kg^−1^ microbial agent, (6) J, 5 mg kg^−1^ microbial agent. Each treatment was replicated three times.

Cultivation Experiment: before the commencement of the cultivation, 50 g of air-dried soil was weighed and placed into a wide-mouth bottle. Deionized water was added to bring the soil moisture content to 60% of its field water-holding capacity. The soil was then pre-incubated at 25°C for 9 days to activate the soil microorganisms (He et al., 2023). After the pre-cultivation period, the biochar and microbial inoculants were added to the corresponding soil treatments, respectively. The mixtures were thoroughly homogenized, and the bottles were sealed with plastic wrap, which was punctured with multiple small holes to maintain aeration. The wide-mouth bottles were then placed in a 25°C incubator for dark incubation for 90 days, with the consumed water being replenished by weighing every 2 to 3 days.

### 2.3 Determination of soil physical and chemical properties

Soil pH was measured using a pH meter (PB-10, Sartorius, USA) at a soil-to-water ratio of 1:2.5 (w/v). Microbial biomass carbon (MBC) and microbial biomass nitrogen (MBN) were determined using the chloroform fumigation method. The measurement steps are as follows: 10 g of fresh soil was placed into a 50 mL centrifuge tube, then extracted with 40 mL of 0.5 M K_2_SO_4_ (shaking at 220 rpm for 30 min), and filtered through quantitative filter paper. Meanwhile, 10 g of soil sample was placed in a beaker and fumigated for 24 h, followed by the same operation. After fumigation, the soil sample was transferred to a 50 mL centrifuge tube for extraction. The filtrate was diluted and acidified, and then the total organic carbon content was measured (Multi N/C 3100, Analytic Jena, Germany). *β*-glucosidase was determined using p-nitrophenyl-*β*-D-glucopyranoside as the substrate, and the hydrolysis of p-nitrophenyl-*β*-D-glucopyranoside produces p-nitrophenol, which is measured colorimetrically (p-nitrophenol colorimetric method). Urease was determined using the phenol-sodium hypochlorite colorimetric method, which analyzes urease activity based on the blue indophenol formed by the reaction of the enzyme-catalyzed product ammonia with phenol-sodium hypochlorite. Acid phosphatase was determined using the sodium phenyl phosphate colorimetric method. All colorimetric methods were measured using a UV spectrophotometer (TU-1950, Persee, China).

### 2.4 High-throughput sequencing

DNA extraction and amplification were carried out in a commercial laboratory (GenePioneer Biotechnologies Co. Ltd., Nanjing, China). Soil DNA was extracted using the PowerSoil® DNA Isolation Kit (Mo Bio Laboratories Inc., Carlsbad, CA, USA) and the FastDNA® SPIN Kit for Soil (MP Biomedicals LLC, Solon, OH, USA). Microbial community structure analysis was performed using standardized pipelines including QIIME2 and DADA2 to generate feature sequences, remove low-quality reads and chimeras, followed by comprehensive statistical analyses and visualization of community composition. DNA (20–30 ng) was amplified using the forward primer 515F (5’-GTGCCAGCMGCCGCGG-3’) and the reverse primer 907R (5’-CCGTCAATTCMTTTRAGTTT-3’), targeting the V4 and V5 hypervariable regions of the 16S rDNA. Soil fungi were amplified using the forward primer ITS-1F (5’-CTTGGTCATTTAGAGGAAGTAA-3’) and the reverse primer ITS-1R (5’-GCTGCGTTCTTCATCGATGC-3’). The libraries were quantified using the Quant-iT PicoGreen dsDNA Assay Kit (Omega Bio-tek, Norcross, GA, USA) on the Qubit fluorometer. The constructed libraries were subjected to 2 × 250 bp paired-end sequencing using the Illumina NovaSeq sequencing system. Alpha diversity was calculated using the QIIME2 software, and comparisons between treatments were made based on the Chao1 index, Shannon index, and Simpson index. All datasets analyzed during this study have been deposited in the Supplementary Dataset S1–S4.

### 2.5 Data analysis

All data in this experiment are presented as the mean ± standard error of three replicates. All figures in the text were created using Origin 2024 software. The experimental data were statistically analyzed using SPSS 26.0 software. One-way analysis of variance (ANOVA) and the Least Significant Difference (LSD) method were used for multiple comparisons between different treatments, to analyze the significance of differences between treatments at the *α* = 0.01 and *α* = 0.05 levels.

## 3 Results

### 3.1 Effects of different amendments on soil physicochemical properties

**Figure 1:**
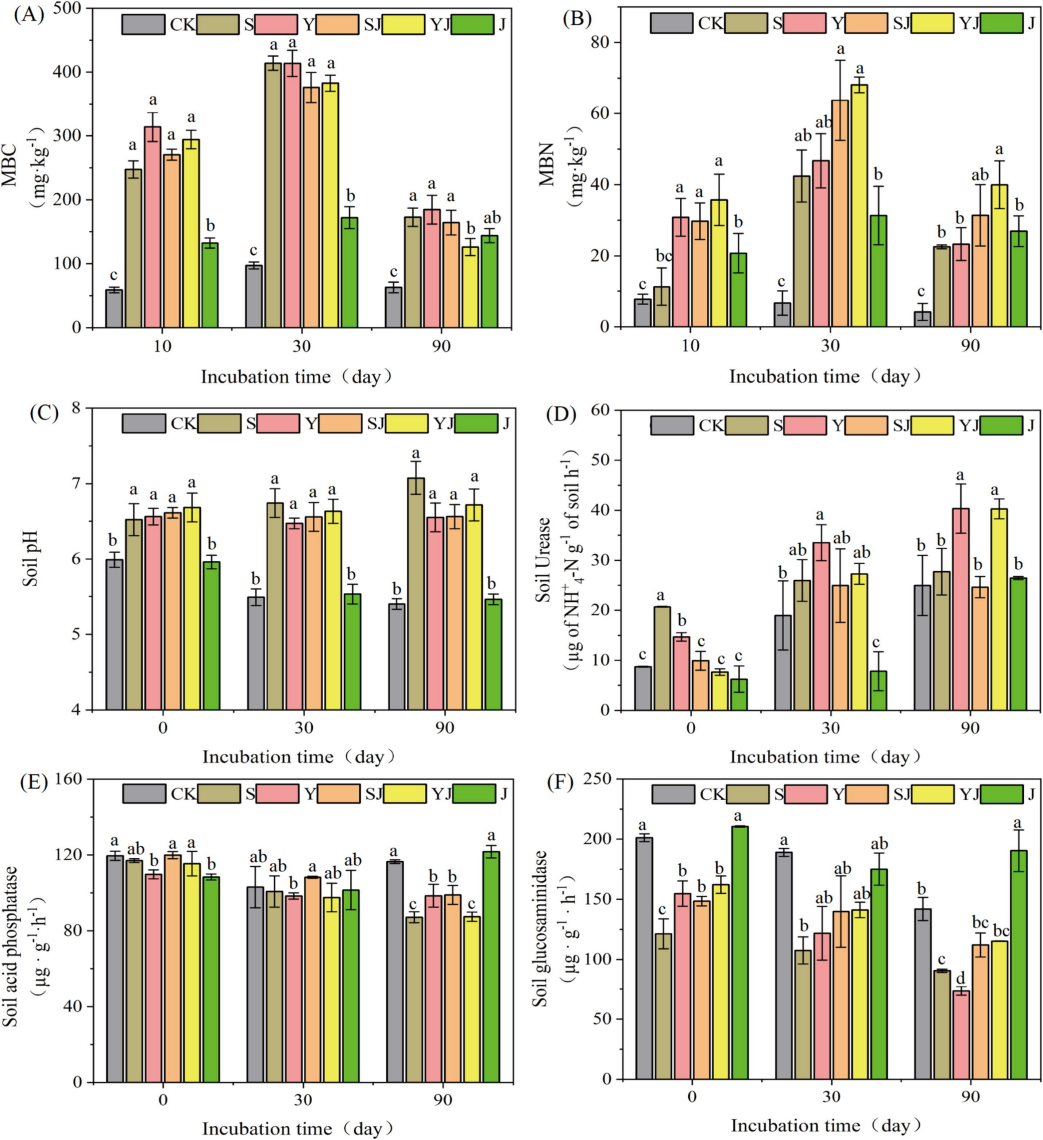
The impact of adding different types of soil amendments on soil physical and chemical properties: **(A)** Microbial Biomass Carbon (MBC) content, **(B)** Microbial Biomass Nitrogen (MBN) content, **(C)** soil pH, **(D)** soil urease activity, **(E)** acid phosphatase activity, and **(F)** glucosidase activity. CK, S, Y, SJ, YJ, and J represent upland soil without any amendment, with rice straw biochar added, with rapeseed straw biochar added, with rice straw biochar + *Bacillus subtilis* agent added, and with rapeseed straw biochar + *Bacillus subtilis* agent added, respectively.

Microbial biomass carbon (MBC) and nitrogen (MBN) represent the assimilated carbon and nitrogen pools within microbial cells, originating from environmental substrates through microbial metabolic processes. These critical biological indicators substantially regulate both the structural composition of soil microbial communities and the transformation rates of key biogeochemical cycles (Liu et al., 2024). As shown in Figure 1A, at the initial stage of cultivation (Day 0), compared to the control (CK), the MBC content in all treatments was significantly increased. Among them, the increase in the Y treatment was the most pronounced, with an increase of 432.39%. In contrast, the MBC content in the J treatment was slightly lower than that in other treatments, only increasing by 124.47% compared to CK (Figure 1A). As the cultivation time was extended, on Day 30, the MBC content in all treatments reached its peak (S: 414.06 mg·kg^−1^, Y: 413.78 mg·kg^−1^, SJ: 376.12 mg·kg^−1^, YJ: 382.74 mg·kg^−1^, J: 171.9 mg·kg^−1^), showing the same trend as on Day 10, that is: the application of biochar or combined microbial agents was more effective than the application of microbial agents alone, and the MBC content in the untreated control was the lowest. By the end of the cultivation period (Day 90), compared to Day 30, the content decreased significantly (by 16.33 to 67.06%), with the most noticeable decrease in the biochar or combined microbial agent treatments (by 55.41 to 67.06%), but still higher than that in the CK. Throughout the cultivation period, the application of biochar, either alone or in combination with microbial agents, resulted in the highest increase in soil MBC. Among the treatments, J exhibited the second-highest MBC content, while CK had the lowest.

As shown in Figure 1B, on the 10th day of cultivation, the MBN content in all treatments was higher than that in the control (CK), and reached its peak on the 30th day of cultivation (CK: 6.66 mg·kg^−1^, S: 42.41 mg·kg^−1^, Y: 46.7 mg·kg^−1^, SJ: 63.73 mg·kg^−1^, YJ: 68.1 mg·kg^−1^, J: 31.34 mg·kg^−1^). Among them, the MBN content in the SJ and YJ treatments was increased by 33.45 and 31.41% compared to the CK, respectively. At the end of the cultivation period (day 90), the MBN content decreased compared to day 30, with a reduction of 14.01 to 50.76%. The order of MBN content was: YJ (39.96 mg·kg^−1^) > SJ (31.38 mg·kg^−1^) > Y (23.32 mg·kg^−1^) > S (22.58 mg·kg^−1^) > J (26.95 mg·kg^−1^) > CK (4.16 mg·kg^−1^). The experimental results demonstrate that the combined application of microbial agents significantly enhances MBN content more effectively than biochar or microbial agents applied individually.

On day 0 of cultivation, compared to the CK, all treatments except for the J group increased the soil pH, with the YJ treatment showing the most significant increase of 0.69 units. At the end of the cultivation period (day 90), the pH of the CK and J treatments dropped to their lowest values of 5.4 and 5.46, respectively. Meanwhile, the S treatment reached a peak value of 7.07, indicating that the addition of biochar alone is more effective in increasing soil pH than the addition of microbial agents alone or in combination, and can more effectively alleviate soil acidification (Figure 1C).

As shown in Figures 1D–F, the activities of Urease, glucosaminidase, and acid phosphatase in upland soil under different treatments are presented. On day 0, compared to the CK group, the soil urease activity was significantly increased (*p* < 0.05) in the S and Y groups at the initial stage of cultivation, with values of S: 20.70 μg of NH^+^_4_-N g^−1^ of soil h^−1^ and Y: 14.69 μg of NH^+^_4_-N g^−1^ of soil h^−1^. On day 30, the treatments with biochar or combined microbial agents increased the soil urease activity by 5.27 to 19.63 μg of NH^+^_4_-N g^−1^ of soil h^−1^, with the Y and YJ treatments showing the largest increases, reaching 18.83 and 19.63 μg of NH^+^_4_-N g^−1^ of soil h^−1^, respectively. Compared to day 30, the urease activity in the Y and YJ treatments was increased to 40.34 μg of NH^+^_4_-N g^−1^ of soil h^−1^ and 40.29 μg of NH^+^_4_-N g^−1^ of soil h^−1^, respectively, on day 90. Meanwhile, at the beginning of the cultivation, different amendment treatments had no significant impact on soil acid phosphatase. At the end of the cultivation, except for the J group, all other amendment treatments decreased the activity of acid phosphatase by 17.56 to 29.3 μg·g^−1^·h^−1^. Throughout the entire cultivation period, the addition of amendments (except for the J group) led to a decrease in glucosidase activity, which slowly decreased with the extension of cultivation time, with the Y group showing the most significant decrease of 73.53 μg·g^−1^·h^−1^, a 31.04% decrease compared to other treatments.

### 3.2 Relative abundance of the soil microbial community

**Figure 2:**
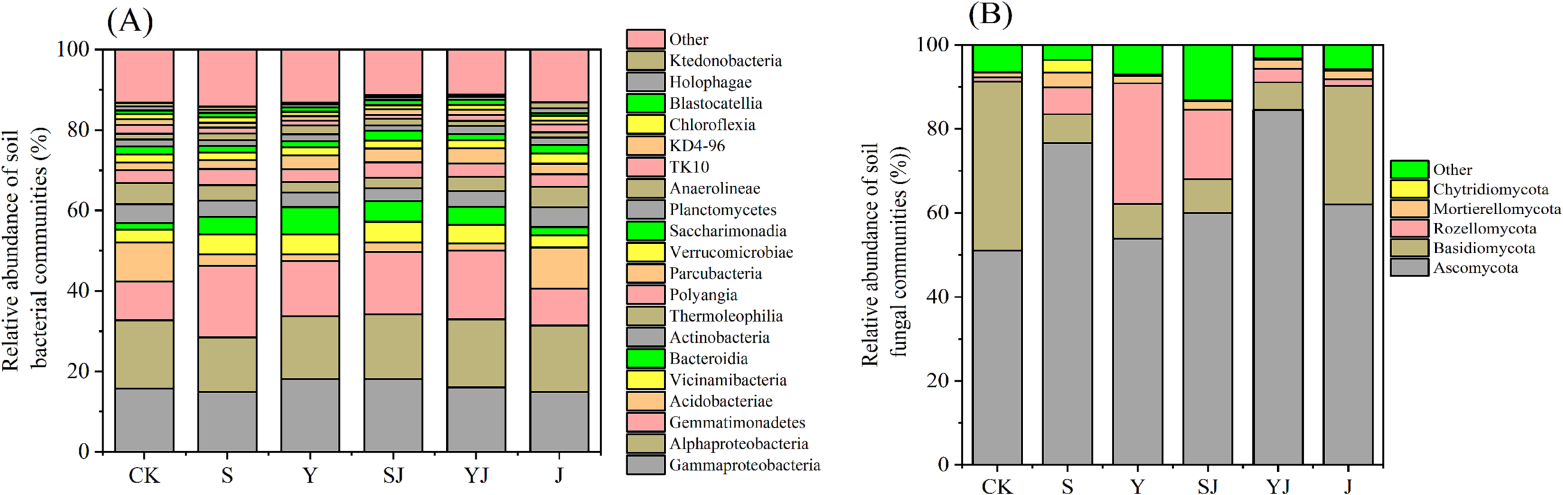
Relative abundance of soil microbial under different amendments. **(A)** Relative abundance of the bacterial community (%) on class level; **(B)** Relative abundance of the fungal community (%) on class level. CK, S, Y, SJ, YJ, and J represent the control, rice straw biochar addition, rapeseed straw biochar addition, rice straw biochar + microbial agent, rapeseed straw biochar + microbial agent, and microbial agent amended soil, respectively.

As shown in Figure 2A, the relative abundance of the main soil microbial groups under different treatments is presented, where the combined proportion of *Proteobacteria*, *Actinobacteria*, and *Acidobacteria* exceeded 50%. The relative abundance of *α-Proteobacteria* and *γ-Proteobacteria* was the highest, reaching 13.6 to 16.9% and 14.8 to 18.1% in all treatment. Except for the J treatment, the relative abundance of *Actinobacteria* was significantly increased (*p* < 0.05) in all amended soils, with the S treatment showing the largest increase, reaching 17.8%, followed by the YJ treatment at 17.0%. In addition, except for the J treatment, the relative abundance of *Acidobacteria* also changed significantly after the addition of amendments, with a substantial decrease of about 70.56 to 82.81%. As shown in Figure 2B, the main fungal community shifts were characterized by the highest proportion of *Ascomycota*, ranging from 51.00 to 84.53%, followed by *Basidiomycota* at 6.57 to 40.29%, and then *Rozellomycota* at 0.99 to 28.72%. Compared to the CK, the application of amendments significantly increased the relative abundance of *Ascomycota* (by 2.85 to 33.53%) and *Rozellomycota* (by 0.58 to 27.73%), while decreasing the proportion of *Basidiomycota* (by 12.09 to 33.71%).

### 3.3 Soil microbial community alpha diversity

**Figure 3:**
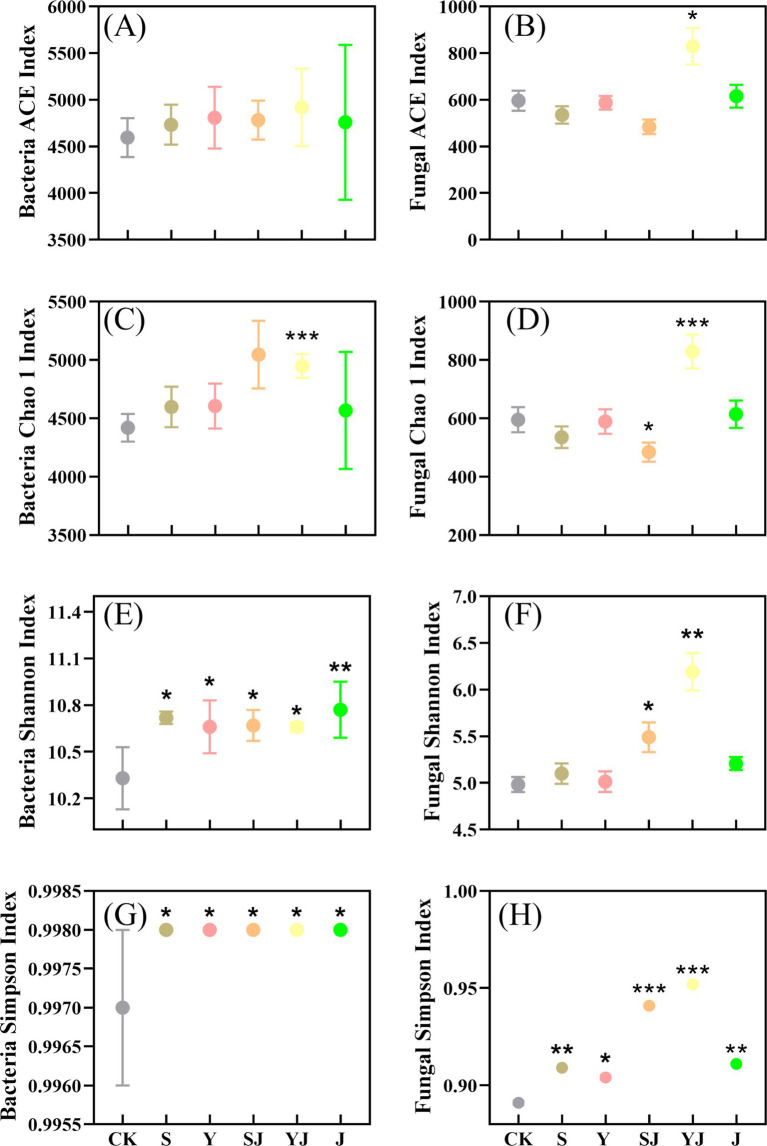
Responses of soil bacterial and fungal community richness and diversity to different treatments, **(A)** represents the Bacterial ACE index, **(B)** the Fungal ACE index, **(C)** the Bacterial Chao 1 index, **(D)** the Fungal Chao 1 index, **(E)** the Bacterial Shannon index, **(F)** the Fungal Shannon index, **(G)** the Bacterial Simpson index, and **(H)** the Fungal Simpson index. (*P < 0.05, **P < 0.01 and ***P < 0.001).

As shown in Figure 3, the changes in the richness and diversity indices of soil bacterial and fungal communities treated with different amendments were presented. The ACE and Chao 1 indices represented the richness of microbial communities, while the Shannon and Simpson indices reflected the diversity of microbial community structures in the soil. Compared to the CK treatment, the ACE index in the S and Y treatments increased by 3.02 and 4.65%, respectively, indicating that the addition of biochar alone enhanced the richness of the soil bacterial community. After the combined use of microbial inoculants, the ACE index showed a slight increase, and the combined treatments had a more pronounced effect on the Chao 1 index, with SJ and YJ treatments showing increases of 9.77 and 7.45% compared to the S and Y treatments, respectively. Compared to the CK, the richness of soil microbial communities in the J treatment only increased by 3.57%. The results showed that the amendments had a certain enhancing effect on the richness of soil bacteria, but there was no statistically significant difference (*p* > 0.05). In addition, all treatments with different amendments significantly increased the Shannon index of soil bacteria (*p* < 0.05), with the J treatment showing the most significant increase (10.77), followed by the S treatment (10.72). Overall, all amendment treatments enhanced the diversity of soil bacteria, but the combined use of biochar and microbial inoculants had a better effect on increasing the richness of soil bacteria compared to other treatments.

The addition of biochar alone led to a reduction in soil fungal richness, ranging from 1.51 to 10.16%. However, when biochar was combined with microbial inoculants, fungal richness increased. Compared to the S treatment, the ACE index in the SJ treatment decreased by 9.53%, while the YJ treatment showed a significant increase (*p* < 0.05), reaching 829.51. Furthermore, while the Shannon index in the S and Y treatments increased compared to the CK, the changes were not statistically significant. Notably, compared to their respective single treatments, the Shannon index in the SJ and YJ treatments increased by 7.65 and 23.5%, respectively. The experimental results indicated that the addition of biochar combined with microbial inoculants enhanced the diversity of soil microbial communities and increased the stability of the soil microbial ecological structure.

### 3.4 Soil microbial community *β*-diversity

**Figure 4:**
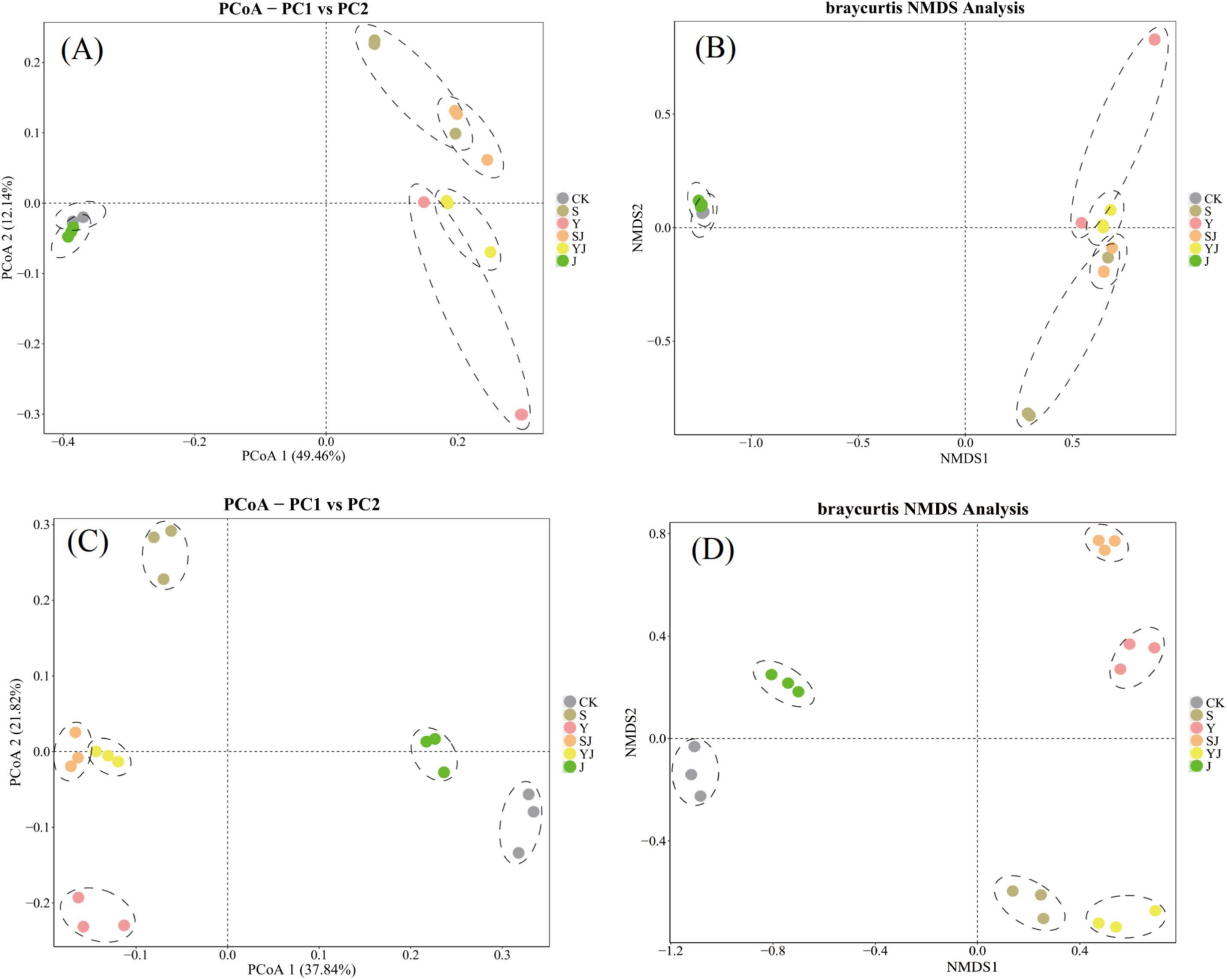
The effects of different amendments on the *β*-diversity of soil microbial communities, **(A)** The PCoA (Principal Coordinates Analysis) of bacterial communities, **(B)** the NMDS (Non-metric Multidimensional Scaling) analysis of bacterial communities, **(C)** the PCoA of fungal communities, **(D)** the NMDS analysis of fungal communities.

In the principal coordinate analysis (PCoA) and non-metric multidimensional scaling analysis (NMDs) of bacterial and fungal communities in dryland soils, samples from the same treatment were closer indicating good repeatability (Figure 4). As shown in Figure 4A, the overlap of CK and J group indicated no significant differences of soil bacteria community between two treatments. The vertical axis separated the CK and J groups from the other treatments. Similarly, in the fungal PCoA, sample distributions mirrored bacterial PCoA patterns, demonstrating pronounced effects of biochar or microbial inoculants on beta diversity for both soil bacteria and fungi. Furthermore, using NMDs analysis to perform a two-dimensional ordering of the bacterial and fungal communities in dryland soils, the results shown significant differences in community structure between the treatments (The stress values are all below 0.1, demonstrating good representativeness.). Among them, the CK and J groups are closely clustered and separated from the other treatments by the vertical axis, showing clear differences. However, compared to the bacterial NMDs analysis, The fungal communities exhibited more pronounced inter-group dispersion patterns in the non-metric multidimensional scaling (NMDs) analysis. This suggested that biochar and microbial inoculants have a stronger influence on bacterial communities than on fungal communities, with the effect being particularly pronounced in the Y group (Figure 4B).

### 3.5 The response of microorganisms to soil environmental factors

**Figure 5:**
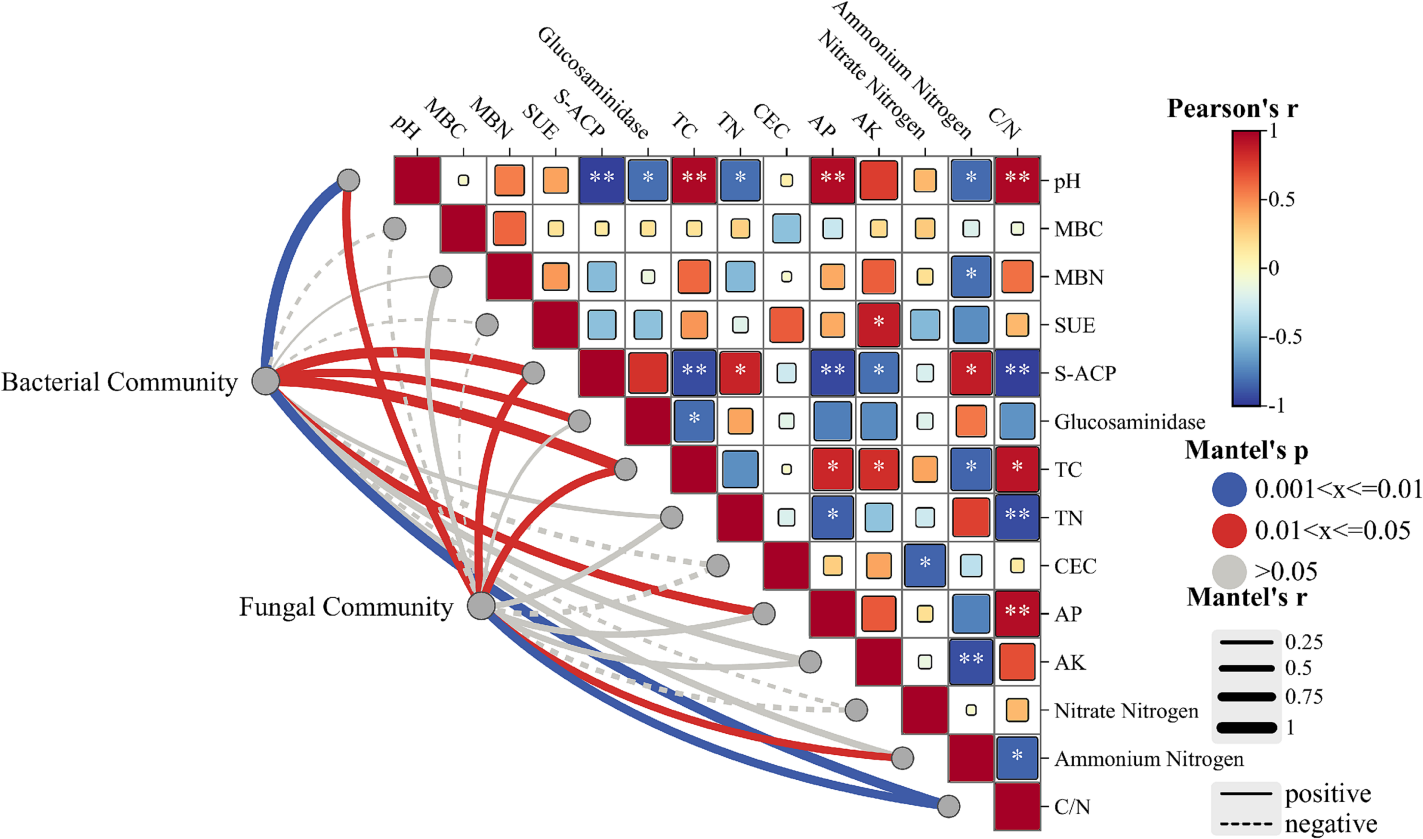
Mantel tests were performed to assess the linkages between soil physicochemical properties and bacterial/fungal community structures.

To further investigate the effects of soil amendments on soil physicochemical properties and microbial community structure, correlation analyses were performed between dominant soil microbial taxa and edaphic parameters (including soil enzyme activities), complemented by Mantel tests assessing bacterial and fungal community linkages (Figure 5). As shown in Figure 5, pH identified as a pivotal environmental factor – exhibited significant negative correlations with acid phosphatase activity (*p* < 0.01), glucosaminidase activity (*p* < 0.05), total nitrogen (TN), and ammonium nitrogen. Conversely, positive correlations were observed between pH and total carbon (TC), available phosphorus (AP), and C/N ratio (*p* < 0.01). Phosphatase activity demonstrated positive associations with TN (*p* < 0.05) and ammonium nitrogen (*p* < 0.01), but negative correlations with TC, AP (*p* < 0.05), available potassium (AK) and C/N ratio (*p* < 0.01). Mantel test results revealed strong soil-microbe linkages: bacterial communities showed significant positive responses to pH (*p* < 0.01), phosphatase activity (*p* < 0.05), glucosaminidase activity, TC, AP, and C/N ratio (*p* < 0.01). Fungal communities were predominantly influenced by pH (*p* < 0.05), phosphatase activity, TC, ammonium nitrogen, and C/N ratio (*p* < 0.01), suggesting these factors serve as key regulators of soil microbial assemblage.

**Figure 6:**
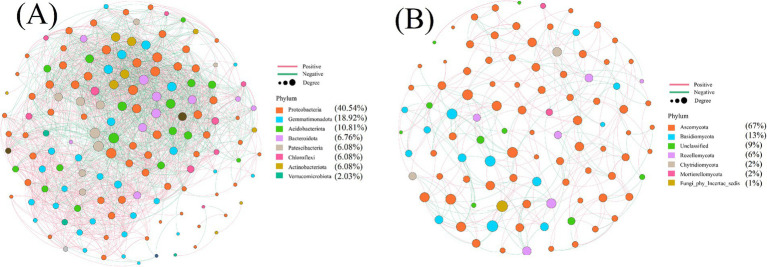
Soil microbial co-occurrence network diagram, **(A)** bacterial community **(B)** fungal community.

In the microbial co-occurrence network analysis, the size of the circles represented the relative importance of each species, as shown in Figure 6. In Figure 6A, *Proteobacteria* dominated, accounting for 40.54%, followed by *Gemmatimonadetes* (18.92%) and *Acidobacteria* (10.81%). In Figure 6B, *Ascomycota* was the most abundant fungal phylum, comprising 67.00%, while *Rozellomycota* represented 6.00%. These taxa not only played a crucial role in the microbial network but also served as key components of the soil microbial community. Furthermore, the relatively high abundance of *Proteobacteria*, *Acidobacteria*, and *Gemmatimonadetes* (collectively exceeding 50%) and *Ascomycota* (ranging from 51.00 to 84.53%) established them as dominant microbial groups in the soil ecosystem.

**Figure 7:**
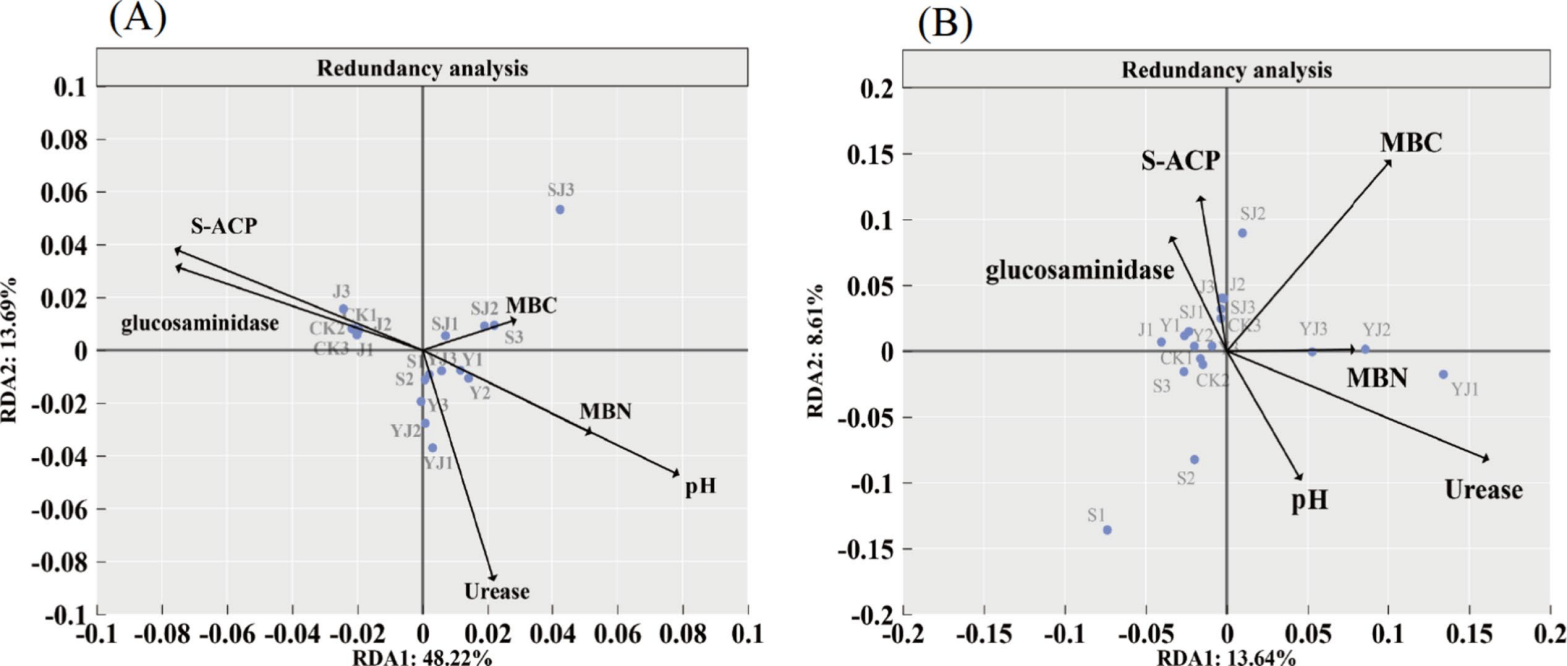
Redundancy analysis, **(A)** bacterial community, **(B)** fungal community.

To further explore the key environmental factors affecting microorganisms, redundancy analysis (RDA) between bacterial and fungal communities and soil environmental factors was conducted (Figure 7). The RDA1 and RDA2 axes explained 48.22 and 13.69% of the variation in the bacterial community, and 13.64 and 8.61% of the variation in the fungal community, respectively. The results showed that in the bacterial community, the interpretation rates of soil environmental factors (pH, MBC, MBN, and urease) were 38.11, 13.94, 25.21, and 11.98%, respectively, while in the fungal community, the interpretation rates were 4.24, 6.96, 5.44, and 11.07%, respectively. Differences in the distribution of treatments were also observed. In Figure 7A, all treatments except the CK and J groups were positioned on the right side of the vertical axis, correlating with pH, MBC, MBN, and urease activity. In Figure 7B, the YJ group was located in the region associated with MBN, suggesting that the YJ treatment had a stronger influence on MBN variations driven by fungal activity. Overall, pH, MBC, and MBN emerged as key environmental factors shaping soil microbial communities, aligning with the findings from the Mantel analysis.

## 4 Discussion

### 4.1 The impact of amendments on soil physicochemical properties and enzyme activities

In this study, the increase in the pH value of dryland soil is one of the important manifestations of the application of amendments, which is consistent with the previous research (Zhang et al., 2019). The increase in soil pH is primarily attributed to the impact of carbonates and ash content generated during the pyrolysis of biomass. In addition, the cations and oxygen-containing functional groups (such as -COOH and -O-) on the surface of biochar can effectively neutralize the H^+^ in the soil solution, further increasing the soil pH. When Yuan and Che (2022) investigated the mechanism of alkaline cations in corn straw biochar on acidic soil, they found that some of the metal cations contained in biochar could reduce the content of exchangeable H^+^ and Al^3+^ in acidic soil, thereby reducing the acidity of the soil. Additionally, the content of MBC and MBN in the soil also increased after the addition of different amendments. The reasons for this phenomenon are, on the one hand, the application of biochar provides many adsorption sites, creating a good habitat for microorganisms, which in turn promotes the growth and reproduction of soil microorganisms and enhances the assimilation of biochar by microorganisms. On the other hand, the application of *Bacillus subtilis* agent increased the producers of MBC and MBN, thereby increasing the microbial biomass. Liang et al. (2017) demonstrated through long-term experiments that sugar and lipids carried in biochar can be effectively utilized by microorganisms. On the other hand, the addition of biochar may affect the synthesis of microbial extracellular enzymes, thereby altering the rates of carbon and nitrogen cycles in the soil (Wei et al., 2021). Liang et al. (2017) pointed out that during the processes of metabolism and assimilation, microorganisms introduce soil carbon sources into the soil through the “microbial carbon pump,” thereby increasing the soil carbon pool. The inoculation with *Bacillus subtilis* promotes the decomposition of recalcitrant carbon through the secretion of organic acids and extracellular enzymes, thereby enhancing the synergistic utilization of N, P by soil microbial communities. However, since microorganisms are extremely sensitive to environmental changes, soil microbial biomass has become an important indicator for assessing soil quality and can thus serve as an early warning indicator for soil microorganisms (López-Aizpún et al., 2018). In this study, both types of biochar used increased MBC and MBN. Hu et al. (2022) found that after applying distiller’s grain biochar to rapeseed-sorghum rotation soil, the soil microbial carbon and nitrogen content significantly increased. Karimi et al. (2020) found that the addition of corn straw biochar increased the microbial biomass carbon in calcareous soil by 1.2 to 2.24 times.

The application of biochar exhibits different responses to soil enzyme activity, which may be related to the porous structure of biochar. This is because its surface and interior can effectively adsorb soil enzymes as well as substrates related to enzymatic reactions. In addition, the nutrients and metal ions provided by biochar may promote the synthesis of specific soil enzymes. In this study, the amendment showed a significant inhibitory effect on acid phosphatase activity at the end of the cultivation period. This result may be attributed to the increase in soil pH value. Previous studies have also indicated that in paddy soils, the application of mushroom biochar led to the inhibition of phosphatase activity due to the increase in soil pH (Sarfraz et al., 2020). Throughout the cultivation period, soil urease activity exhibited an overall increasing trend. However, a significant rise in urease activity was observed only at the end of the experiment in the treatment groups receiving rapeseed straw biochar and complex microbial agents alone, compared to the control (CK). This effect may be attributed to the lower C/N ratio of rice straw biochar relative to rapeseed straw biochar, suggesting a higher availability of nitrogen in rice straw biochar. In the rapeseed straw biochar treatment, microorganisms likely secreted more urease to break down urea and nitrogen-containing organic matter in the soil, fulfilling their nitrogen requirements. In addition, glucosidase, by cleaving glycosidic bonds, catalyzes the conversion of short cellulose oligosaccharides or cellobiose into utilizable glucose in the soil (Salgado et al., 2018). In this study, the treatment groups receiving either biochar alone or the compound microbial inoculant exhibited a continuous decline in *β*-glucosidase activity throughout the incubation period. This phenomenon may be associated with the fact that biochar application provides more readily degradable carbon sources compared to soil polysaccharides, thereby temporarily suppressing *β*-glucosidase activity in the short term. The study by Liu X. et al. (2022) and Liu J. X. et al. (2022) demonstrated that *β*-glucosidase activity exhibited positive correlations with methoxy groups, phenolic compounds, and alkyl compounds following long-term incubation. Notably, biochar amendment significantly enhanced soil *β*-glucosidase activity, which further corroborates the conclusion.

### 4.2 The impact of amendments on soil bacterial and fungal communities

As illustrated in Figure 3, the application of biochar and *Bacillus subtilis* enhanced the richness of soil bacterial communities, as reflected by the ACE and Chao 1 indices, while fungal richness was generally suppressed, except in the YJ treatment. Nevertheless, all treatments contributed to an overall increase in the diversity of both bacterial and fungal communities. This effect can be attributed to the synergistic influence of biochar, which elevated soil pH, enriched organic matter content, and improved the soil ecological condition (Zhao J. et al., 2024). Furthermore, biochar contains essential trace elements such as K, Ca, and Mg, which are crucial for microbial growth. Its intricate porous structure provides a suitable habitat for microorganisms, effectively shielding them from environmental stressors (Cole et al., 2019). In summary, the combined application of biochar and *Bacillus subtilis* leads to an increase in soil microbial community richness. This phenomenon can be attributed to the porous structure and adsorption sites of biochar, which provide a suitable environment for *Bacillus subtilis*, facilitating its secretion of extracellular enzymes to accelerate organic matter decomposition. This process supplies additional carbon sources for other microorganisms. Additionally, the interaction may also involve quorum sensing mechanisms that regulate the metabolic activities of both *Bacillus subtilis* and surrounding microbes, thereby promoting symbiotic relationships (Rather et al., 2021).

As demonstrated by Singh et al. (2022) in a comprehensive review of 59 studies investigating variables such as biochar feedstock, pyrolysis temperature, and pyrolysis duration, the addition of biochar significantly enhances soil bacterial diversity. This enhancement is closely associated with increases in total carbon (TC) and total nitrogen (TN) in the soil. Furthermore, in their investigation of plant resistance to environmental stress, Liu J. X. et al. (2022) discovered that compounds such as N-acyl homoserine lactones (AHLs) and coumaric acid can enhance inter-microbial communication. Additionally, the water retention capacity of biochar plays a beneficial role in facilitating the transmission of signaling molecules. The enhanced richness and diversity of soil microbial communities are conducive to improving the buffering capacity of soil microecology against environmental fluctuations and stress resistance. This enhancement crucially depends on the strategic combination of labile carbon and recalcitrant carbon, coupled with simultaneous provisioning of resources for both copiotrophic and oligotrophic microorganisms.

In this study, the combined application of biochar and microbial inoculants had a pronounced impact on soil bacterial communities, notably influencing *Proteobacteria*, *Gemmatimonadetes*, and *Acidobacteria*. Specifically, the relative abundance of *Proteobacteria* and *Gemmatimonadetes* exhibited a significant increase, whereas *Acidobacteria* showed a marked decline. These aligning with Yang et al. (2022) could be attributed to bacterial community responses to changes in soil physicochemical properties. This phenomenon further indicates an increase in soil nutrient content following the addition of the amendments. Among these, *Proteobacteria*, as copiotroph microorganisms, play a significant role in soil carbon cycling and are widely distributed in soil environments (Liu et al., 2020). *α-Proteobacteria* and *γ-Proteobacteria* play crucial roles in the soil ecosystem by degrading toxic and harmful substances. They are particularly significant in the biodegradation of pollutants such as polycyclic aromatic hydrocarbons (PAHs), which are generated during the pyrolysis process of biochar (Haleyur et al., 2019). Based on this, the treatment strategy of applying biochar in combination with microbial agents can not only significantly enhance the nutrient cycling efficiency of the soil, but also effectively improve the soil tolerance and remediation capacity for harmful pollutants. Moreover, the addition of biochar significantly increased the soil pH value, leading to a marked downward trend in the relative abundance of *Acidobacteria*. However, in the treatment with the application of microbial agents alone, although the abundance of *Acidobacteria* increased to some extent, it did not reach a statistically significant level. This phenomenon may be due to *Bacillus subtilis* in the soil decomposing organic matter, releasing extracellular polysaccharides and organic acids, thereby reducing the soil pH value, and thus promoting the growth and reproduction of *Acidobacteria* and other acidophilic bacteria (Redmile-Gordon et al., 2020). In the bacterial community, in addition to the significant changes in *Acidobacteria*, the relative abundance of *Gemmatimonadetes* increased due to the application of biochar or composite microbial agents (Figure. 2), which was consistent with the research conclusions of Chen et al. (2023) and Deng et al. (2022). *Gemmatimonadetes* is a phosphorus-accumulating bacterium. The addition of biochar to the soil can promote the synthesis of readily available phosphorus, thereby increasing the relative abundance of *Gemmatimonadetes* (Su et al., 2015).

In addition to soil bacteria, the fungal community is also an important component of the soil microbial environment. The experimental results show that the addition of biochar and microbial agents significantly increased the diversity of soil fungi, which is crucial for maintaining the stability of the soil microbial ecosystem. This effect may be attributed to the incomplete combustion products generated during the pyrolysis process of biochar, which provide suitable energy sources and environmental conditions for the growth of saprotrophic fungi (Zhang et al., 2021). In this study, the addition of biochar and microbial agents promoted the relative abundance of *Ascomycota* and *Rozellomycota*, while reducing the relative abundance of *Basidiomycota*. This result was consistent with the research conducted by Yang et al. (2024). Han et al. (2023) shown that the addition of biochar shifted core composition of the soil fungal network from *Basidiomycota* to *Ascomycota*. The increase in the relative abundance of *Ascomycota* may be related to the increase in soil pH caused by the addition of amendments, as *Ascomycota* prefer a neutral environment. Notably, rice straw biochar and rape straw biochar amendments differentially enhanced *Ascomycota* abundance in soil, potentially attributable to the smoother surface topography of rice straw biochar that facilitated hyphal network proliferation in this fungal phylum. Furthermore, *Ascomycota* can enhance the soil resistance to environmental stress (Sarkar et al., 2021). After the application of biochar or the combined use of biochar with *Bacillus subtilis*, the relative abundance of *Basidiomycota* decreased. This is attributed to the fact that *Basidiomycota* primarily decomposes lignin in the soil (Manici et al., 2024), and the recalcitrant carbon in biochar is difficult for it to effectively decompose and utilize. Moreover, the combination with *Bacillus subtilis* may increase spatial competition, hindering the colonization and growth of *Basidiomycota*. Our results indicated that the application of biochar and microbial agents could increase the relative abundance of beneficial organisms (such as *Gemmatimonadetes* and *Ascomycota*), enhance the capacity to cope with environmental stress, and optimize the soil micro-ecosystem. It held significant importance for the structure of soil microbial communities.

Future research will enhance the exploration of how microbial communities impact soil nutrient cycling and delve deeper into the mechanisms through which microbes influence soil nutrients. Laboratory-derived incubation protocols will be scaled to field implementations, though recognizing that field conditions fundamentally differ from controlled laboratory settings through temperature fluctuations, precipitation variability, and anthropogenic disturbances (e.g., tillage-induced soil structural modifications). Enhanced monitoring regimes will prioritize thermohydrological parameters to better align experimental interpretations with natural ecosystem processes.

## 5 Conclusion

This study demonstrates that the application of biochar and *Bacillus subtilis* preparations significantly influences microbial biomass, enzyme activity, and soil microbial community structure in dryland soils. Specifically, biochar application notably increased the relative abundance of *Proteobacteria, Gemmatimonadetes, Ascomycota,* and *Rozellomycota* while reducing *Acidobacteria* and *Basidiomycota*. Among the treatments, rapeseed straw biochar and its composite microbial agents exhibited the most pronounced effects. Additionally, different amendments enhanced bacterial richness and diversity in dryland soil. In the short term, the composite amendment treatment led to a significant increase in microbial biomass carbon (MBC) and microbial biomass nitrogen (MBN), whereas long-term observations suggested that applying the microbial agent alone resulted in more stable effects. Furthermore, rapeseed straw biochar and its composite microbial agents significantly boosted soil urease activity, though their impact on acid phosphatase activity was negligible. Overall, these amendments optimized the soil microbial community structure, contributing to soil improvement, increased crop yields, and enhanced ecological stability. However, future research should further investigate the influence of soil salinity, water content, and heavy metal availability on the evolution of soil microbial communities. This study provides a valuable scientific foundation for understanding the impact of biochar and composite microbial agents on dryland soil microbiota, offering important practical implications for sustainable soil management.

## Sample availability

Samples of the compounds are available from the authors.

## Data Availability

The original contributions presented in the study are included in the article/Supplementary material, further inquiries can be directed to the corresponding author/s.
