# Supplementary material for: Biochar and Bacillus subtilis co-drive dryland soil microbial community and enzyme responses
Source: Front Microbiol. 2025 May 27;16:1603488. doi: 10.3389/fmicb.2025.1603488 (PMC12149193; doi:10.3389/fmicb.2025.1603488)
Supplement: Supplementary file 1 [file Data_Sheet_1.doc]

**Appendix A Supplementary data**

**Supporting Information A1** Biochar Preparation Process.

**Supporting Information A2** Soil culture operations.

**Supporting Information A3** Methods for the determination of soil enzyme activities.

**Supporting Information A4** Relevant data on soil microorganisms.

**Supporting Information A1** Biochar Preparation Process.

The oilseed rape straw and rice straw were mechanically crushed and subsequently passed through a 60-mesh sieve. The resulting powder was placed in a tube furnace, where pyrolytic carbonization occurred at 600 ℃ in a nitrogen atmosphere, with a heating rate of 5 ℃ per minute for a duration of 2 hours. After the carbonization process, the material was cooled, removed from the furnace, and ground to pass through a 100-mesh sieve. The ground powder was then dried at 105 ℃ until a constant weight was achieved, following multiple washings with distilled water. Finally, the biochar was stored in a sealed container for future use.

**Supporting Information A2** Soil culture operations.

The different experimental treatments were incubated at a constant temperature of 25 ℃ to ensure soil aeration over a 90-day dark incubation period. The soil was rehydrated every two days to maintain a moisture content equivalent to 60% of the water holding capacity (WHC). Soil samples collected throughout the experiment were analyzed for physicochemical properties and biological activity.

**Supporting Information A3** Methods for the determination of soil enzyme activities.

The *β*-glucosidase (S-*β*-GC) was measured by taking 0.05 g of air-dried soil sieved through 50 mesh sieve and adding 25 µl of toluene and shaking at room temperature for 15 min, then adding 400 p-nitrophenyl-*β*-D-glucopyranoside and sodium acetate buffer pH 5.5, shaking at 37 ℃ for 1 h and then immediately after the reaction in a water bath at 90 ℃ for 5 min, cooled by running water and then centrifuged at 25 ℃ at 10000 g for 10 min and then measured the absorbance at 400 nm. The absorbance was measured at 400 nm by centrifugation at 10000 g for 10 min. Urease (S-UE) was detected by taking 0.25 g of fresh air-dried soil samples and adding 125 µl of toluene and shaking it well to make the soil samples wet all over, and then adding 625 µl of urea and 1250 µl of sodium citrate buffer (pH=6.7) after leaving it at room temperature for 15 min, and then placing the samples at 37 ℃ for 24 h and then incubating them at 25 ℃, After centrifugation at 10000 g for 10 min, the supernatant was diluted 10 times, and then 400 µl of the diluted supernatant was mixed with sodium benzoate and sodium hypochlorite, and then the absorbance was measured at 578 nm to calculate the urease activity after resting for 20 min; Acid phosphatase (S-ACP) was measured by taking 0.1 g of air-dried soil samples, adding 50 µl of toluene and shaking for 15 min, and adding disodium benzoate and shaking the sample for 24 h at 37 ℃.

**Supporting Information A4** Relevant data on soil microorganisms.

The experimental data is provided as required. In accordance with the research group's regulations, we are unable to provide the complete dataset. Selected critical data has now been compiled into an Excel file.
